# High Piezoelectric Conversion Properties of Axial InGaN/GaN Nanowires

**DOI:** 10.3390/nano8060367

**Published:** 2018-05-25

**Authors:** Nikoletta Jegenyes, Martina Morassi, Pascal Chrétien, Laurent Travers, Lu Lu, Francois H. Julien, Maria Tchernycheva, Frédéric Houzé, Noelle Gogneau

**Affiliations:** 1 Centre de Nanosciences et de Nanotechnologies—CNRS-UMR9001, Université Paris-Sud, Université Paris-Saclay, F91120 Palaiseau, France; jegenyes@free.fr (N.J.); martina.morassi@u-psud.fr (M.M.); laurent.travers@u-psud.fr (L.T.); lu.lu@c2n.upsaclay.fr (L.L.); francois.julien@u-psud.fr (F.H.J.); maria.tchernycheva@u-psud.fr (M.T.); 2 Laboratoire de Génie Électrique et Électronique de Paris, UMR 8507 CNRS-Centrale-Supélec, Université Paris-Sud, Université Paris-Saclay et UPMC-Sorbonne Université, F91190 Gif-sur-Yvette, France; pascal.chretien@geeps.centralesupelec.fr (P.C.); frederic.houze@geeps.centralesupelec.fr (F.H.)

**Keywords:** III-N nanowires, piezoelectric generation, atomic force microscope, piezo-generators, energy harvesting

## Abstract

We demonstrate for the first time the efficient mechanical-electrical conversion properties of InGaN/GaN nanowires (NWs). Using an atomic force microscope equipped with a modified Resiscope module, we analyse the piezoelectric energy generation of GaN NWs and demonstrate an important enhancement when integrating in their volume a thick In-rich InGaN insertion. The piezoelectric response of InGaN/GaN NWs can be tuned as a function of the InGaN insertion thickness and position in the NW volume. The energy harvesting is favoured by the presence of a PtSi/GaN Schottky diode which allows to efficiently collect the piezo-charges generated by InGaN/GaN NWs. Average output voltages up to 330 ± 70 mV and a maximum value of 470 mV per NW has been measured for nanostructures integrating 70 nm-thick InGaN insertion capped with a thin GaN top layer. This latter value establishes an increase of about 35% of the piezo-conversion capacity in comparison with binary p-doped GaN NWs. Based on the measured output signals, we estimate that one layer of dense InGaN/GaN-based NW can generate a maximum output power density of about 3.3 W/cm^2^. These results settle the new state-of-the-art for piezo-generation from GaN-based NWs and offer a promising perspective for extending the performances of the piezoelectric sources.

## 1. Introduction

The number of nomad micro-devices for medical implants, sensing, monitoring and personal electronics is constantly rising in our daily life and the development of autonomous power supply systems constitutes an important challenge. Today, the most common solution for self-supplying micro-systems consists in using batteries. Although the recent developments of the batteries have led to an improvement of their energy density [1,2], these sources are limited by their complex integration, their limited capacity and their cost, to be used to supply micro-devices. In this context, the development of new energy generation technologies relying on ultra-compact and integratable sources and generating sufficient power to supply the micro-devices without increasing their weight is vital for sustainable, independent and maintenance-free operation of micro-devices.

Small-scale energy harvesting is a promising perspective to make the micro-devices energy-autonomous. Indeed, the direct conversion of ambient renewable energies into electrical energy, offers an elegant solution to supply embedded system without the need of grid connection or batteries. Among the alternative ambient energy resources, the mechanical vibrations and deformations (such as body movements, sound vibrations, hydraulic movements, wind, friction …), that can be harvested using piezoelectric materials, present the advantages in many environments to be ubiquitous, available at all time and more accessible than solar and thermal energy.

In the last decade, thanks to the break-through in the synthesis of nanomaterials, 1D piezoelectric nanostructures (such as nanowires (NWs), nanorods, nanofibers …) have emerged as excellent candidates to fabricate novel ultra-compact and highly efficient piezo-generators, then opening new application fields, such as the powering of wireless micro-devices. Their attractiveness stems from their superior mechanical properties [3,4], higher sensitivity to applied force [4,5] and higher piezoelectric coefficients [6,7,8] in comparison to conventional 2D films and bulk materials.

Since the first demonstration of electrical generation from the lateral bending of ZnO NWs in 2006, then defining for the first time the nanogenerator concept [9], other 1D-nanostructures have been investigated such as CdS [10], CdSe [11], PZT [12], BaTiO_3_ [13], KNBO_3_ [14] … These last years, the attention has turned to III-nitride NWs thanks to their high-piezoelectric coefficients [15] and their strong piezo-generation response. The first mechanical-electrical conversion from GaN nanorods has been reported in 2007 [16]. But it is only starting from 2010, that III-nitride 1D-nanostructures are investigated as potential nanomaterials for piezoelectric generation. To our best knowledge, the best mechanical-electrical conversion in terms of output voltage has been obtained with III-Nitride NWs. Hence, a maximum generated voltage of about −440 mV [17] and 1 V [18] have been reported for n-doped GaN and InN NWs, respectively. These outputs illustrate the high potential of nitride 1D-nanostructures for developing efficient piezo-generators, since they largely exceed those for other piezoelectric nanostructures and especially ZnO NWs which are today the most studied nanomaterials for piezo-conversion.

The studies of the piezoelectric properties of individual 1D-nanostructures have naturally lead to the fabrication of macroscopic piezo-generators with different designs and integrating various 1D-nanostructures such as PVDF nanomaterials [19,20,21], PZT nanofibers [22], ZnO [23,24] or III-nitride NWs [25,26,27]. While these energy sources can generate power densities in the μW-mW/cm^3^ range, their use as micro-system power supplies is rather limited, especially due to their poor energy conversion. Indeed, to reach these generation capacities, devices of several square centimetres in size, and/or the application of pressures of the order of several MPa are required, not allowing their use as viable integrated energy sources. To consider the NW-based energy harvesting systems for supplying micro-devices, the improvement of the conversion capacity and efficiency of the piezoelectric active layer is a pre-requisite to the extension of the generator performances.

In this work, we demonstrate that the energy generation capacity and efficiency of GaN nanowires can be enhanced by integrating an In-rich InGaN section in the NW volume. Using an atomic force microscope (AFM) equipped with a modified home-made Resiscope module [17], we systematically investigate the piezo-conversion properties of individual molecular beam epitaxy (MBE) grown p-doped In_0.35_GaN_0.65_/GaN NWs in a dense array. We establish that the piezoelectric response of InGaN/GaN heterostructure NWs can be tuned as a function of the InGaN insertion thickness and its position in the NW volume (i.e., the InGaN insertion is localized at the top or in the volume of the GaN NW). We also evidence that the energy harvesting is more efficient when InGaN insertion is capped with a thin GaN layer since the PtSi/GaN Schottky nanocontact, through which are collected the generated piezo-charges, leads to an improved conductance in comparison with PtSi/InGaN Schottky one. Hence, we report average output voltages up to ~330 ± 70 mV and a maximum value of 470 mV per NW integrating 70 nm-thick InGaN insertion capped with a thin GaN top layer. This result constitutes the first demonstration of mechanical-electrical conversion from axial InGaN/GaN NWs and highlight an enhancement of about 35% of the piezo-conversion capacity in comparison with non-heterostructured binary p-doped GaN NWs (maximum output voltage of 350 mV per NW [28]). By considering the maximum output signals, we have estimated a maximum output power of 223 pW per NW and a power density generated by one layer of InGaN/GaN NWs of about 3.3 W/cm^2^. These maximum powering capacities estimated from AFM measurements on free-standing NWs demonstrate the impact of InGaN insertion to enhance the energy generation and thus offer a promising perspective for the exploitation of InGaN/GaN heterostructured NWs as active media for high-efficient ultra-compact piezo-generators.

## 2. Materials and Methods

**Figure 1:**
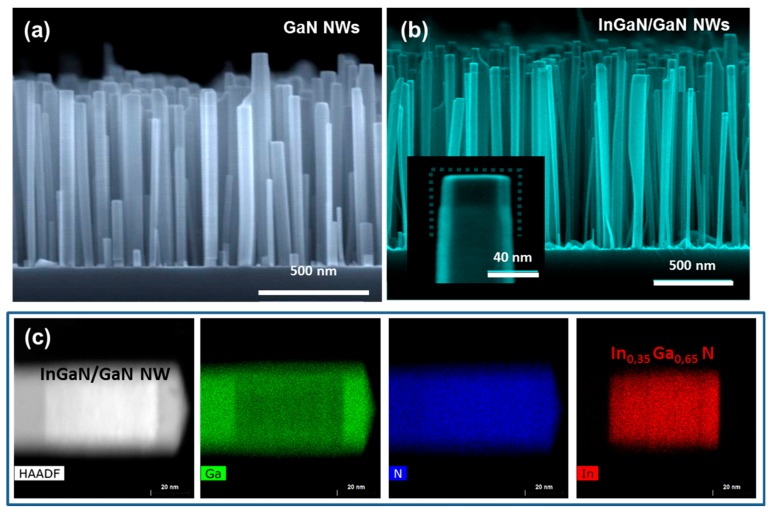
Scanning electron microscope images of (**a**) GaN and (**b**) InGaN/GaN nanowires (NWs) grown by plasma-assisted molecular beam epitaxy (MBE). (**c**) Transmission electron microscope image and Ga, N and In energy-dispersive X-ray spectroscopy (EDX) mappings of the top part of a capped InGaN/GaN NW.

Self-assembled InGaN/GaN NWs were grown on conductive oxide-free Si (111) substrates (resistivity of the order of 0.007 Ω cm) in a molecular beam epitaxy chamber (MBE) (Riber, Bezons, France), equipped with a radio-frequency N plasma source. Prior to the growth of InGaN/GaN NWs, a 2.5-nm-thick AlN buffer layer was deposited on the substrate following a previously reported procedure [29], to allow for a better control of the NW nucleation, density and orientation [30,31,32]. The GaN NW bases were grown at 790 °C under N-rich conditions with an N/Ga flux ratio of 1.36 (Figure 1a). Then, the substrate temperature was ramped down to 590 °C under growth interruption to grow disk-shaped axial In_x_Ga_1−x_N heterostructures with an average In content of x = 0.35 ± 0.05 (Figure 1b) within the In-adlayer growth regime [33]. Following these growth conditions, we obtained highly homogeneous In-rich In_0.35_Ga_0.65_N insertions as illustrated by the high angle annular dark field scanning transmission electron microscopy (HAADF-STEM) (FEI, Hillsboro, OR, United States) and energy-dispersive X-ray spectroscopy (EDX) mappings (FEI, Hillsboro, OR, United States) of Figure 1c. In addition, in some samples, a GaN cap was grown on top under N-rich conditions (N/Ga ratio of 25) at 650 °C to preserve abrupt InGaN/GaN interfaces.

The resulting InGaN/GaN NWs are vertically aligned with hexagonal cross-section shape delimited by {10-10} planes [34]. The NWs are also characterized by N-polar top surface (NWs grown along the [000, 1] direction), which implies that under compressive strain the resulting piezoelectric potential created inside the nanostructures is positive, while under tensile strain the potential is negative as explaining in detail in ref. [35].

**Figure 2:**
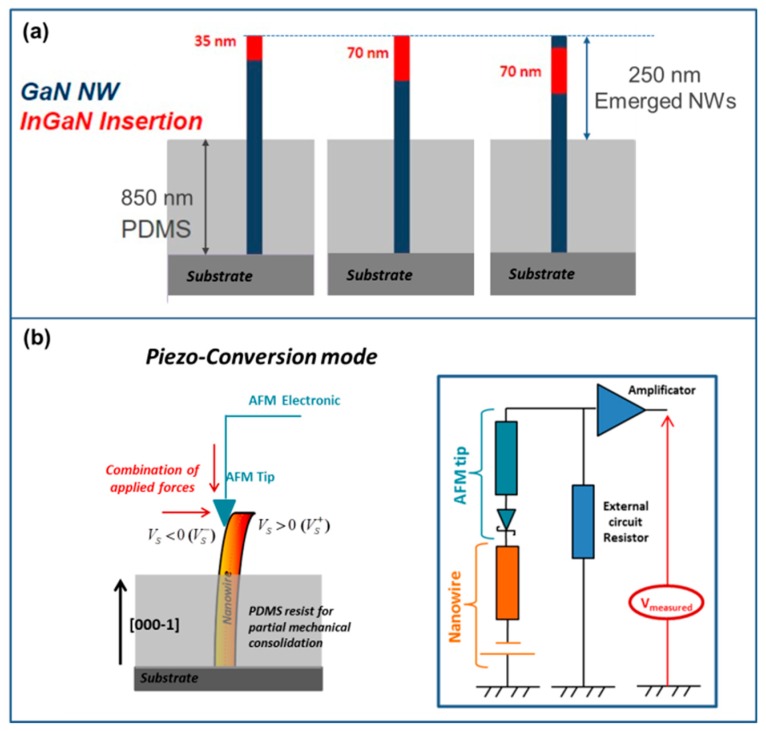
(**a**) Schematization of the investigated structures: GaN NW base with an InGaN insertion localized on the top. From left to right: uncapped 35 ± 5 nm thick InGaN section; uncapped 70 ± 9 nm thick InGaN section and capped 70 ± 9 nm thick InGaN section. (**b**) Schematic representation of the piezo-generation measurement principle on single NWs, using an atomic force microscope (AFM) system in vertical configuration equipped with a modified Resiscope module. To perform accurate piezo-conversion measurements under AFM, the NWs have been mechanically consolidated with an 850 nm thick PDMS matrix, the emerged NW height being of the order of 250 nm.

In the present work, three different sets of samples were investigated, as schematized on Figure 2a. Two sets consisted of InGaN/GaN NWs with a single uncapped InGaN insertion (of 35 ± 5 nm and 70 ± 10 nm thickness respectively) localized on the GaN NW top (scanning electron microscope (SEM) micrograph (FEI, Hillsboro, OR, United States) of Figure 1b); while the third set had a 70 ± 10 nm thick InGaN section capped with a 10 nm-thick GaN layer (Figure 1c). For each series, the NWs are characterized by a length of 1.1 ± 0.1 μm, a diameter of 50 ± 20 nm and a density of the order of 1.5 × 10^10^ NW/cm^2^.

In the present work, three different sets of samples were investigated, as schematized on Figure 2a. Two sets consisted of InGaN/GaN NWs with a single uncapped InGaN insertion (of 35 ± 5 nm and 70 ± 10 nm thickness respectively) localized on the GaN NW top (scanning electron microscope (SEM) micrograph (FEI, Hillsboro, OR, United States) of Figure 1b); while the third set had a 70 ± 10 nm thick InGaN section capped with a 10 nm-thick GaN layer (Figure 1c). For each series, the NWs are characterized by a length of 1.1 ± 0.1 μm, a diameter of 50 ± 20 nm and a density of the order of 1.5 × 10^10^ NW/cm^2^.

In order to measure accurately the piezoelectric response of individual NWs (with the AFM system described below) and because the III-N NWs are characterized by a high flexibility, the NWs have been mechanically consolidated by embedding their bottom portion in an 850 nm thick polydimethylsiloxane (PDMS) layer (Figure 2a). This flexible matrix allows ensuring the mechanical consolidation of the NWs without applying to them any deformation or strain (as verified by Raman spectroscopy measurements non-presented here). The height of the emerging NW portions is of about 250 ± 100 nm, while the density of the NWs remains unchanged.

The piezoelectric conversion properties of the NWs have been investigated by using an atomic force microscope (Bruker Nano Surface, Palaiseau, France) in the vertical configuration equipped with a modified Resiscope module [17]. This method combines the nanometric scale resolution of the AFM and the real-time electrical measurement of the Resiscope [36] allowing resistance measurements over a ten decades range (10^2^–10^12^ Ω) in the dynamic conditions of AFM imaging. In this experimental configuration, the AFM tip scans over the surface of the NWs array in contact mode and under a controlled and constant normal force. The AFM tip induces a lateral deflection of the nanostructure, which results in the appearance of an asymmetric strain across the NW, from a tensile strain to the outer face of the NW to a compressive one to the inner face. As a consequence, a piezoelectric field is created inside the NW evolving respectively from negative to positive value [35] as schematized in Figure 2b. The electrical response of the NW is monitored by the modified home-made Resiscope). This electrical module is connected to the substrate (via an ohmic contact formed between the NW bottom and the substrate [37]) and to the conductive AFM tip. The used PtSi AFM tip being characterized by a work function of about 5 eV [38] and the GaN by an electron affinity of the GaN is 4.1 eV, the AFM tip/GaN NW contact forms a Schottky diode, through which are collected the piezo-generated charges [39]. In our specific AFM configuration, the topographic and the electrical signals are continuously recorded. No external voltage is applied during the scanning, and the outputs piezo-generated by the NWs are recorded through a load resistance of 1 GΩ. This experimental technique allows characterizing the piezo-conversion properties of single NWs over a large surface, which provides statistic measurements.

## 3. Results

**Figure 3:**
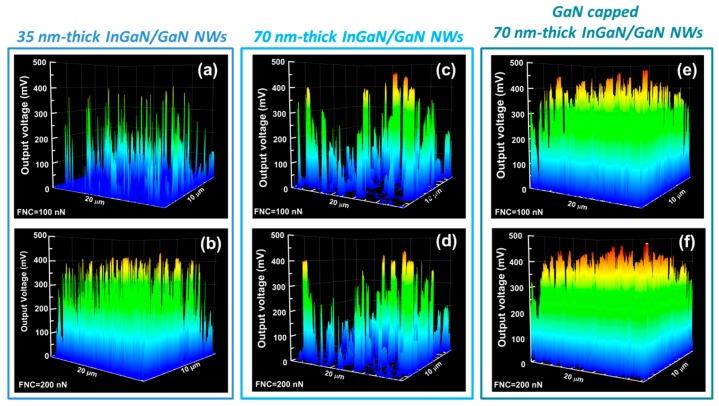
3D output voltage mappings collected on (**a**,**b**) 35 nm-thick InGaN/GaN; (**c**,**d**) 70 nm-thick InGaN/GaN; and GaN capped 70 nm-thick InGaN/GaN NWs (**e**,**f**) using an AFM equipped with a modified Resiscope module in piezo-generation configuration for two different constant normal forces of 100 and 200 nN.

Figure 3 presents 3D mappings of output voltage peaks generated by the three sets of InGaN/GaN NW arrays recorded for constant normal force (CNF) of 100 and 200 nN. (Intermediate CNF values of 30, 60 and 150 nN were also tested but corresponding images are not presented for the sake of length). It must be mentioned that the CNF is much higher than the one (few nN) used for characterizing the free-standing ZnO [9] and other 1D-nanostructures by using equivalent AFM equipment. In fact, as detailed before, the NWs are mechanically consolidated due to their high flexibility. In consequence, a higher deflection force is needed to bend the emerging NWs and thus reach the same degree of deformation as the free-standing nanostructures. In the electrical mappings, each peak corresponds to the piezoelectric response of an individual NW and exhibits a positive output voltage. This is in agreement with the p-type conductivity of the NWs induced by using Magnesium during their growth [28,40]. Results presented in Figure 3 constitute the first demonstration of piezoelectric conversion from InGaN/GaN-based NWs.

**Figure 4:**
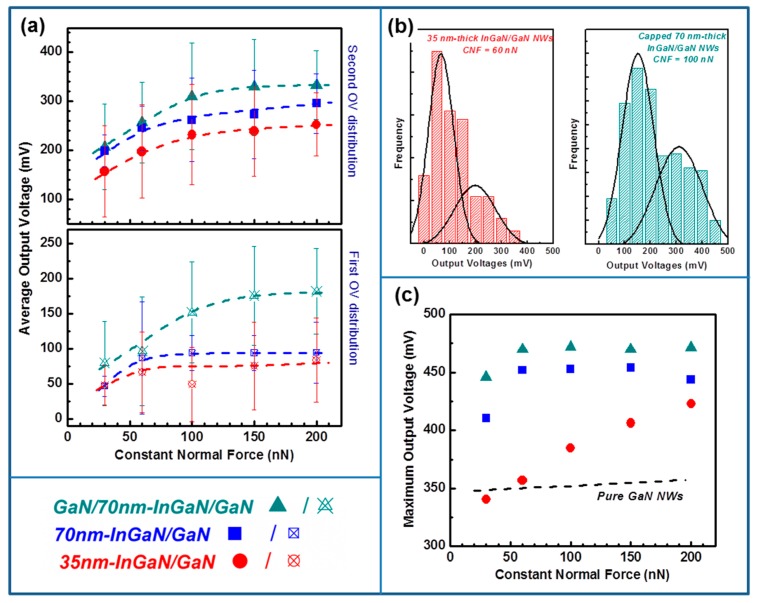
(**a**) Average and (**c**) maximum output voltages measured on InGaN/GaN NWs as a function of the constant normal force for the three sets of samples. For comparison, the maximum output voltages generated by non-heterostructured binary p-doped GaN NWs (from [28]) are also represented by (**c**). (**b**) Histogram distribution of the output voltages generated by 35 nm-thick uncapped InGaN/GaN and 70 nm-thick capped InGaN/GaN NWs respectively for constant normal force (CNF) of 60 and 100 nN.

The maximum and average output voltages are presented in the Table 1 and in Figure 4 for each set of samples as a function of the CNF. The output voltages statistics showed a bimodal distribution, well approximated by two Gaussian functions. The error bars in Table 1 correspond to half-width at half maximum of the corresponding Gaussian fits.

The piezoelectric response of NWs strongly depends on their flexibility, which itself depends on the NW dimensions. In the present case, the height of the NWs emerging from the PDMS matrix is about 250 nm ± 100 nm (Figure 2a). The small protruding NWs (height up to 150–200 nm) are characterized by a high rigidity which limits their deformation by bending and thus the creation of an efficient piezoelectric potential inside the nanostructure. By contrast, for higher NWs, the flexibility being more important, it is easier to reach a higher piezoelectric-field, then allowing the generation of a larger output voltage. In our experimental configuration, since each mapping has been realized in the same scanning conditions (i.e., with the same scanning rate and constant normal force), the output voltage distribution directly illustrates the flexibility dispersion of the NWs. We can thus attribute the double dispersion of measured output voltages (illustrated in Figure 4b for two statistics) to the height dispersion of the NWs, which originates from the self-assembled growth mode used to synthesis them.

For the three sets of samples, we can clearly observe an increase of the output voltages with the constant normal force, in agreement with experimental results [17,28,41] and theoretical predictions [42]. This behaviour is explained by the convolution of two phenomena: (i) the increase of the piezoelectric potential with the increase of the NW deflection; (ii) and the improvement of the Schottky contact stability with increasing force applied by the conductive AFM tip [38,43] and thus allowing a better harvesting efficiency.

Our results also evidence that the insertion of InGaN section in the volume of p-doped GaN NWs induces an improvement of piezoelectric response of the NWs in comparison with binary p-doped GaN NWs. This behaviour is well illustrated on Figure 4c, where the maximum output voltages generated by the different sets of samples, as well as the measured ones for binary p-doped GaN NWs [28], have been plotted as a function of the constant normal force. The observed saturation of the generated outputs can be explained by a saturation of the internal electric field inside the NWs, resulting from the saturated rotation of electric dipoles [44], and/or by a limitation of the harvested energy through the Schottky contact, as we have recently reported [38].

For the highest applied CNF (200 nN), the piezo-conversion measurements assessed by AFM yield average output voltages per NW in the 253–333 mV range and maximum ones in the 423–472 mV range. These latest values largely exceed the maximum voltages generated by other families of piezoelectric 1D-nanostructures (PZT, CdS, CdSe …) and especially ZnO NWs (max. 90 mV per NW [45]), the widely investigated nanostructures for developing piezo-generators. Especially, for GaN-capped 70 nm-thick InGaN/GaN NWs, the maximum output reaches 470 mV per NW, then demonstrating an improvement of approximately 35% of their piezo-conversion capacity in comparison with p-doped GaN NWs [28,38], as well as an improvement by 7% in comparison with the highest reported voltage for single n-doped GaN NWs [17]. This result settles thus the new state-of-the-art for piezoelectric generation from GaN-based NWs and demonstrates the high potential of InGaN/GaN NWs for developing efficient generators.

**Figure 5:**
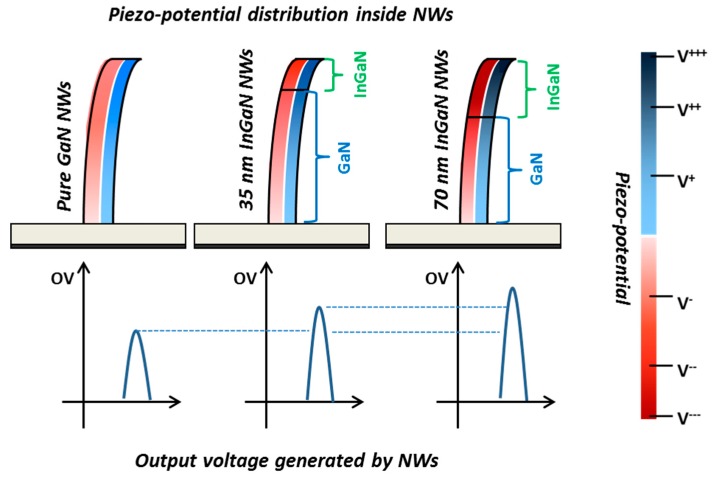
Schematization of the piezo-potential distribution as a function of the NW hetero-structuration and the corresponding output voltage generated for binary GaN NWs (**left side**), 35 nm-thick InGaN/GaN NWs (**middle**) and 70 nm-thick InGaN/GaN NWs (**right side**). The thicker is the InGaN insertion, the higher is the piezo-potential and the larger is the output voltage.

The integration of one InGaN insertion in the GaN NW volume results unambiguously in an improvement of its generation capacity. At this stage, one may wonder which mechanism is in play. The InGaN material is characterized by higher piezoelectric coefficients in comparison with GaN [46]. An equivalent degree of deformation will thus induce the appearance of a more important piezoelectric field inside the NW containing InGaN and thus the generation of a higher output voltage. This behaviour is confirmed if we analyse the results from the point of view of the InGaN insertion thickness (35 nm-thick and 70 nm-thick InGaN/GaN NWs). In fact, as the insertion thickness increases, the piezo-generated output voltages also increase (Figure 4). The InGaN insertion is integrated at the NW top, where the deformation is more pronounced. The increase of the piezo-generated energy is thus more important when the InGaN insertion is thick and thus the locally created piezoelectric field is higher. This behaviour is experimentally confirmed by the different conversion efficiencies observed in Figure 4 between the uncapped 35 nm-thick and 70 nm-thick InGaN/GaN NWs. For thinner insertion, the maximum output voltages increase quasi-linearly with applied force, while for the thicker one, the outputs saturate for constant normal force larger than 60 nN. This evolution of the generated signals illustrates lower conversion efficiency in the case of the 35 nm-thick insertions which results from the creation of a smaller piezoelectric field in comparison with the thicker insertion, for an equivalent deformation force. Figure 5 schematizes this mechanism by illustrating the piezoelectric field distribution and the corresponding output voltages for binary GaN and uncapped 35 nm-thick InGaN/GaN and 70 nm-thick InGaN/GaN NWs.

If now, we compare the piezo-generation of uncapped and GaN-capped 70 nm-thick InGaN/GaN NWs (Figure 3 and Figure 4), one may wonder why the presence of a GaN cap layer leads to an improvement of the piezo-response of the system. The measured output voltage depends mostly on the capacity of the piezoelectric material (here the NWs) to efficiently convert the mechanical deformation into electrical energy. However, it also strongly depends on the efficiency of the metal/semiconductor electrode to harvest the piezo-generated energy. By capping with GaN the 70 nm-thick InGaN insertion, we have modified the characteristics of the Schottky diode formed between the PtSi AFM tip and the NW. We have recently demonstrated that because the AFM tip/NW contact is governed by its nanometre size, the conventional description of the Schottky diode cannot be applied [38]. In the case of Schottky nanocontact, the effective Schottky barrier height becomes a function of the diode size. In this condition, the contribution of the tunnelling emission becomes dominant and thus gives rise to an improved energy harvesting [47,48].

**Figure 6:**
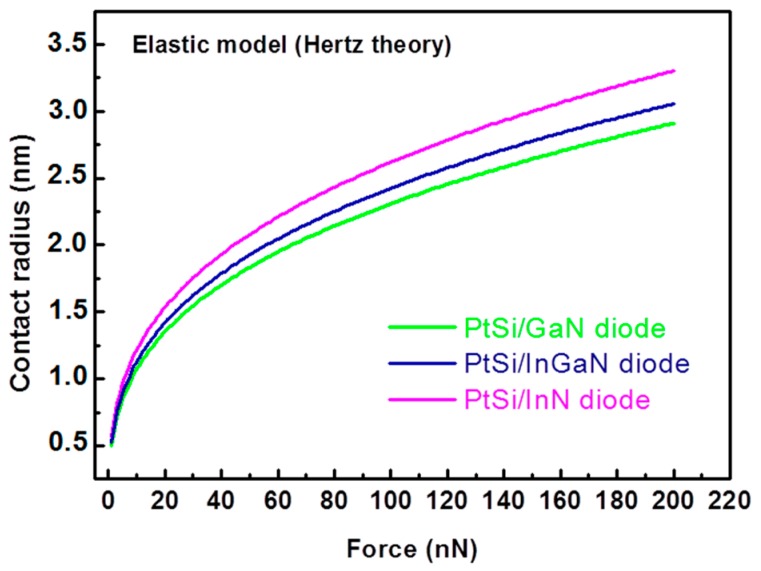
Calculation of the contact radius between PtSi AFM tip and GaN (green line), InN (pink line) and InGaN (blue line) NWs as a function of the applied force. The Hertz theory has been used with the material setting from Table 2.

The diode size is directly related to the AFM tip radius (which is of 25 nm for PtSi AFM tip [38]) and to the properties of the material constituting the top surface of the NW. We have calculated the contact surface between the PtSi AFM tip and the InGaN, GaN and InN NW top by using the Hertz theory [38,49]. This theory describes a regime of purely elastic deformation between two perfectly smooth solids, in the absence of adhesion and friction. In these conditions, the mechanical contact between a sphere of radius R (here the AFM tip radius) and a plane (here the GaN top NW surface) is expressed as a disk of radius *a* by the following equation: a=(34 F RE*)13, where *F* is the applied force, *R* is the AFM tip radius and E* is the reduced Young’s modulus of the two materials and given by $\frac{1}{E^{*}}\;=\;\left(\frac{1-\nu_{1}^{2}}{E_{1}}\right)+\;\left(\frac{1-\nu_{2}^{2}}{E_{2}}\right)$ with *E*_1_, *E*_2_ and *ν*_1_, *ν*_2_ are respectively the Young’s modulus and Poisson’s ratios of the two materials. The materials parameters considered for the calculations are presented in the Table 2. The InGaN parameters have been calculated by considering the Vegard’s Law with the elastic parameters of bulk GaN and InN [50] and an In content of 35%. To illustrate our approximation of the InGaN parameters, we have represented on Figure 6 the contact radius for PtSi/InN interface.

Figure 6 presents the calculated contact radius for both uncapped and GaN-capped InGaN/GaN NWs as a function of the constant normal force. We can observe that whatever the Schottky diode, the contact radius, which is extremely small (of about few nm), is lower than the depletion width in III-N NW, which typically varies between 20 and 80 nm depending on the dopant density and/or the NW diameter [56,57,58]. This result confirms the nanocontact behaviour of the Schottky diode. Then, the smaller is the diode size, the thinner is the barrier and the higher is the conductance. Because at a given force the contact radius is smaller for the GaN/PtSi interface than for the InGaN/PtSi one, the harvested energy is increased. This result is consistent with the increased output voltages measured for the capped NWs under nominally identical CNF.

The experimental results presented in this work demonstrate that the piezoelectric conversion of GaN NWs is substantially enhanced by the presence of axial InGaN heterostructures. Hence, the integration of these nanostructures appears as a promising solution for improving the piezoelectric generator performances. In order to illustrate this high potential, we have estimated the capacity of the three sets of NW samples for powering nanodevices. To determine the maximal output power density generated by our p-doped InGaN/GaN NWs, we have considered the following relation: $P=\frac{\bar{V_{i}^{2}}}{R_{L}}\;\cdot \rho$, where $V_{i}$ is the output voltage generated by the *i*-th NW, $R_{L}$ is the resistance across which the measurement is performed ($R_{L}=1\;G\Omega$) and ρ is the surface density of NWs. Assuming that each NW produces the maximal output voltage for a constant normal force of 200 nN and that the NW density was about 1.5 × 10^10^ NW/cm^2^, we have estimated and reported in Table 3 the maximum output power and the maximum power densities for the three sets of samples. The estimated values are, respectively, in the 179–223 pW per NW and in the 2.7–3.3 W/cm^2^ ranges. These values, to the best of our knowledge, exceed all previously reported ones assessed by AFM technique. Regarding the high powering capacity of these free-standing, the InGaN/GaN-based NWs present high potentialities for developing integratable and high-efficient piezoelectric energy harvested sources.

## 4. Conclusions

In summary, we have demonstrated for the first time the high piezoelectric response of InGaN/GaN-based NWs. Using an AFM equipped with a modified Resiscope module, we have shown that it is possible to tune the piezo-conversion properties of individual MBE-grown p-doped In_0.35_GaN_0.65_/GaN NWs by modulating the InGaN insertion thickness and position in the NW volume. Based on piezo-response assessed by AFM and on calculation of the Schottky diode size using the Hertz theory, we have evidenced that the conductance at the PtSi/GaN Schottky nanocontact is more favourable in comparison to the PtSi/InGaN one and thus yields an improvement of the piezoelectric harvesting. Hence, under lateral deformation of the NWs, an average output voltage up to ~330 ± 70 mV and a maximum value of 470 mV per NW integrating a 70 nm-thick InGaN insertion capped with a thin GaN top layer have been reported. This latter value is larger by 35% in comparison with p-doped GaN NWs (maximum output voltage of 350 mV per NW [28]) and larger by 7% in comparison with the highest reported voltage for single n-doped GaN NWs (maximum output voltage of −442 mV per NW [17]). This result settles thus the new state-of-the-art for piezoelectric generation from GaN-based NWs and demonstrates the high potential of InGaN/GaN NWs for developing efficient generators. By considering the maximum output signals measured by AFM on free-standing NWs, we have estimated a maximum output power of 223 pW per NW and a power density generated by one dense layer of InGaN/GaN NWs of about 3.3 W/cm^2^. These results demonstrate the capability of InGaN insertion to enhance the energy generation and thus offering a promising perspective to develop ultra-compact and high-efficient renewable energy harvesters for powering micro-devices.

## Figures and Tables

**Table 1:** Average values, standard deviations and maximal output voltages (with a measurement precision of 2%) generated by NWs as a function of the constant normal force.

Constant Normal Force (nN)	Average Output Voltage/Standard Deviation (mV) *Double Distribution of the Output Voltages*			Maximum Output Voltage (mV)		
	35 nm-InGaN	70 nm-InGaN	Capped 70 nm-InGaN	35 nm-InGaN	70 nm-InGaN	Capped 70 nm-InGaN
**30**	*48 ± 29* *158 ± 93*	*46 ± 15* *199 ± 33*	*79 ± 60* *208 ± 87*	*340*	*411*	*446*
**60**	*66 ± 58* *198 ± 95*	*86 ± 80* *246 ± 45*	*96 ± 78* *257 ± 82*	*357*	*452*	*470*
**100**	*49 ± 53* *232 ± 102*	*94 ± 25* *263 ± 86*	*152 ± 72* *310 ± 109*	*385*	*453*	*472*
**150**	*75 ± 62* *239 ± 92*	*93 ± 25* *273 ± 90*	*175 ± 71* *330 ± 96*	*406*	*454*	*470*
**200**	*84 ± 60* *253 ± 64*	*94 ± 43* *296 ± 61*	*182 ± 61* *333 ± 70*	*423*	*444*	*472*

**Table 2:** Material settings used for the calculations (* refer to bulk values).

	GaN	InGaN	InN	PtSi
**Young’s modulus *E*** **(GPa)**	300 * [51]	**244**	149 * [52]	**238** [53]
**Poisson coefficient *ν***	**0.4** [54]	**0.25**	**0.365** [55]	**0.32** [53]

**Table 3:** Maximum output voltages, output powers and power densities of 35 nm-thick InGaN/GaN; 70 nm-thick InGaN/GaN; and GaN capped 70 nm-thick InGaN/GaN NWs at a constant normal force of 200 nN.

Constant Normal Force (nN)	Maximum Output Voltage (mV)			Maximum Output Power (pW per NW)			Maximum Power Density (W/cm²)		
	35 nm-InGaN	70 nm-InGaN	Capped 70 nm-InGaN	35 nm-InGaN	70 nm-InGaN	Capped 70 nm-InGaN	35 nm-InGaN	70 nm-InGaN	Capped 70 nm-InGaN
**200**	*423*	*444*	*472*	*179*	*197*	*223*	*2.7*	*3*	*3.3*
